# From Local Issues to Global Impacts: Evidence of Air Pollution for Romania and Turkey

**DOI:** 10.3390/s24041320

**Published:** 2024-02-18

**Authors:** Tugce Pekdogan, Mihaela Tinca Udriștioiu, Hasan Yildizhan, Arman Ameen

**Affiliations:** 1 Department of Architecture, Faculty of Architecture and Design, Adana Alparslan Türkeş Science and Technology University, Adana 46278, Turkey; tpekdogan@atu.edu.tr; 2 Department of Physics, Faculty of Sciences, University of Craiova, 200585 Craiova, Romania; mtudristioiu@central.ucv.ro; 3 Department of Energy Systems Engineering, Adana Alparslan Türkeş Science and Technology University, Adana 46278, Turkey; hyildizhan@atu.edu.tr; 4 Department of Building Engineering, Energy Systems and Sustainability Science, University of Gävle, 801 76 Gävle, Sweden

**Keywords:** air pollution, statistical analysis, monitoring, PM sensors, ecosystems

## Abstract

Air pollution significantly threatens human health and natural ecosystems and requires urgent attention from decision makers. The fight against air pollution begins with the rigorous monitoring of its levels, followed by intelligent statistical analysis and the application of advanced machine learning algorithms. To effectively reduce air pollution, decision makers must focus on reducing primary sources such as industrial plants and obsolete vehicles, as well as policies that encourage the adoption of clean energy sources. In this study, data analysis was performed for the first time to evaluate air pollution based on the SPSS program. Correlation coefficients between meteorological parameters and particulate matter concentrations (PM_1_, PM_2.5_, PM_10_) were calculated in two urban regions of Romania (Craiova and Drobeta-Turnu Severin) and Turkey (Adana). This study establishes strong relationships between PM concentrations and meteorological parameters with correlation coefficients ranging from −0.617 (between temperature and relative humidity) to 0.998 (between PMs). It shows negative correlations between temperature and particulate matter (−0.241 in Romania and −0.173 in Turkey) and the effects of humidity ranging from moderately positive correlations with PMs (up to 0.360 in Turkey), highlighting the valuable insights offered by independent PM sensor networks in assessing and improving air quality.

## 1. Introduction

Climate change generates high-temperature variations and extreme weather phenomena, causing health issues, especially for sensitive individuals with lower thermoregulation capabilities. Climate change creates difficulties for the cardiopulmonary and gastrointestinal systems and increases the risk of infectious and allergic diseases [1]. Within climate change, urban heat and air pollution contribute to the rise in temperature [2,3].

Particulate matter (aerosol particles/solid particles) represents one of the main categories of air pollutants, together with gaseous pollutants (SO_2_, NO_x_, CO, O_3_, VOC), Polycyclic Aromatic Hydrocarbons (PAHs), persistent organic pollutants (dioxins), and heavy metals (Pb, Hg). It is an important proxy indicator for air pollution. The primary sources of PM_10_ (aerodynamic diameter smaller than 10 μm) are represented by motor vehicle exhaust emission and tire abrasion, resuspended soil [4], industrial pollution and dust emission, and biomass burning [5]. Millán-Martínez et al. (2021) [6] classify the primary sources of PMs as natural and anthropogenic. Sources of PM_2.5_ include nitrate and sulfate, biomass burning, crustal material, vehicle emissions and road dust, wood combustion, vegetative detritus, secondary aerosol, fossil fuel combustion, traffic exhausts, and cooking factors [7,8,9]. The chemical characteristics of PM_1_ are concentrations of water-soluble inorganic ions, organic and elemental carbon, and mineral dust [10], and primary sources are represented by ammonium, nitrate, sulfate, chloride, and organic [11]. Other authors noted that sources of PM_1_, including ammonium nitrate and secondary inorganic aerosol (road traffic, domestic heating, biomass burning), peaked in the winter. The summer season also increased marine components and mineral dust [12].

The harmful effects of air pollution on human health are discussed in many studies. PM_10_ primarily accumulates in the upper respiratory tract. However, fine (PM_2.5_ and PM_1_ account for small particles of less than 2.5 and 1μm in diameter, respectively) and ultra-fine (PM_0.1_) particles can reach lung alveoli [13,14]. Regarding cardiovascular [15,16] and respiratory [17,18,19] effects, fine and ultrafine particles are more dangerous than PM_10_ [20]. The impacts on human health might vary from nausea [21] to difficulty breathing [22] and lung cancer [23,24,25]. Air pollution affects pregnant women, children’s development, and older people with comorbidities because it reduces their immune system activity [26]. Zhu et al. (2021) [27] show that air pollution significantly increases people’s resistance to antibiotics.

Stroke mortality and hospital admissions are more likely when people are exposed to PM_1_, PM_2.5_, and PM_10_ for a short time. Moreover, PM_1_ has a stronger association with ischemic stroke incidence than PM_2.5_ and PM_10_ [28]. Ma et al. (2023) [29] showed that PM_1_ and PM_2.5_ increase myocardial infarction mortality and the incidence of cardiorespiratory diseases [30]. Lu et al. (2023) [31] emphasize the effect of PM_1_, PM_2.5_, and PM_10_ on influenza-like illness.

Higher PM_1_ exposure has been linked to dyslipidemias and blood lipids. Older, heavier, or male participants are more susceptible to PM_1_’s adverse effects [32]. Also, long-term exposure to PM_1_ leads to hypertension and blood pressure [33], incident asthma among middle-aged and elderly adults [34], metabolic syndrome [35], and lung function in children and adolescents [36].

Short-term PM_2.5_ exposure increases the risk of acute nasopharyngitis [37], while long-term exposure to PM_2.5_ leads to neurological disorders [38].

The WHO decreased the alert criteria in 2021 to lessen the negative impacts of air pollution on human health [39]. The threshold for PM_10_ is 45 µg/m^3^, and for PM_2.5_, it is 15 µg/m^3^ (24 h of exposure; no more than three or four exceedances per year). For PM_1_, there are no recommendations for the moment.

The European Union has an ambitious European Green Deal, which aims to reduce net greenhouse gas emissions by at least 55% by 2030, compared to 1990 levels, to make Europe the first continent with no net emissions of greenhouse gases by 2050. The European Commission agreed to continue improving air quality standards in the EU, bringing them more in line with WHO [39] guidelines. This concept was included in the zero-pollution action plan, which established a goal for 2050 of lowering air, water, and soil pollution to levels no longer deemed hazardous to human health and ecological systems, thus contributing significantly to the environmental goals of the wider Eurasian continent.

Report 5/2022 of the European Environmental Agency established air quality standards for 12 pollutants. The aim is to protect human health and the environment. For PM_2.5_, the limit is 20 µg/m^3^, with an average period of one year; for PM_10_, it is 50 µg/m^3^, with an average period of 24 h, 35 days of exceedances permitted each year, and 40 µg/m^3^ for an average period of one year [40]. The zero-pollution action plan sets a vision for reducing pollution levels in the EU for 2050 “to levels no longer considered harmful to health and natural ecosystems.” Moreover, this plan introduced essential targets for 2030 to reduce the health impacts of air pollution by more than 55% compared to 2005. In October 2022, the European Commission proposed Euro 7 emission standards for road vehicles.

In 2020, Romania had the highest EU health cost, EUR 1810 per capita [41,42], and in 2021, Turkey’s health expenditure per capita was TRY 4206 [43].

It is in the power of local decision makers to monitor and reduce air pollution in their communities because they cannot neglect health and environmental costs. The money to solve these issues might be invested in modern technologies, green areas, non-polluting heat systems, and infrastructure. These decision makers have the power to control industrial facilities and to develop policies to reduce air pollution in urban areas. Moreover, it is in the power of communities to check the information delivered by their leaders. Regarding air pollution, the local community can develop independent sensor networks for air quality monitoring, primarily when national agencies do not communicate well. Citizen science can potentially transform the community’s understanding of environmental problems. Also, the universities that are changing vectors in their areas can develop projects, sensor networks, national and international academic collaborations to solve local problems, and awareness campaigns to help citizens understand the importance of clean air for their health.

This work introduces a project result and presents the usefulness and importance of expanding a local PM sensor network at the international level. Velea et al. (2020) [44] conducted a statistical analysis of air pollution in eight Romanian cities, including Craiova, based on the Copernicus Atmosphere Monitoring Service regional ensemble modeling system. They compared their results with measured data available from the European Environment Agency. Moreover, the authors underlined the advantages of independent sensor networks as an alternative data source in areas where PM measurements are unavailable. The current study reveals a new facet of statistical analysis of air pollution research by presenting the first comparison of air quality measurements between Adana/Turkey and Drobeta-Turnu Severin and Craiova/Romania. This paper sheds light on air pollution in these regions and emphasizes the importance of internationally expanding local PM sensor networks. By rigorously analyzing quarterly data covering meteorological parameters and particulate matter concentrations, this study provides convincing evidence that urgent action is needed to combat air pollution and its harmful effects on human health. The findings underline the need to monitor air quality, implement effective policies, and utilize modern technologies to reduce the adverse effects of air pollution. This study, therefore, serves as a clarion call to researchers, policymakers, and community leaders worldwide to prioritize air quality improvement strategies and accelerate efforts to create cleaner and healthier environments for all. In addition, this study pinpoints the air quality in cities from two countries, thus pleading for the importance of such independent sensor networks in air pollution monitoring. The advantage of such an independent sensor network is that it has better spatial coverage of air pollution due to a larger number of sensors than the official network. The disadvantage is given by measuring PM concentration (laser scattering) compared with the official stations of the national environmental agencies (gravitational). Nevertheless, the laser scattering method underestimates PM concentrations, and the independent network might complement the official one, mainly when the official network does not produce data or as a double check for air pollution.

## 2. Data and Methods

**Figure 1:**
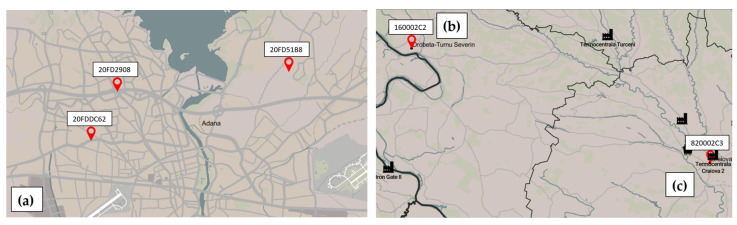
PM sensors’ locations in (**a**) Adana/Turkey (20FDDC62, 20FD2908 and 20FD51B8), (**b**) Drobeta-Turnu Severin/Romania (160002C2), and (**c**) Craiova/Romania (820002C3).

This section briefly describes how the sensors’ locations were chosen and the features of each city’s climate. In this work, we used five sensors, three located in Adana, Turkey, and two in Craiova and Drobeta-Turnu Severin, Romania as shown in Figure 1. The sensors used in this study can measure temperature, relative humidity, and PM_1_, PM_2.5_, and PM_10_ concentrations. The sensors from Adana were made by students during summer school using a kit of sensors equivalent to PM Smoggie. Adana has the size and population of Bucharest, the capital of Romania. Five sensors were used in this study, three in Adana/Turkey and one each in Craiova and Drobeta-Turnu Severin/Romania. The distribution of sensors was strategically planned. With its higher population density and relatively few environmental monitoring stations, Adana required a denser sensor network to adequately capture spatial variability in air quality [45,46]. This strategic positioning allows for a more comprehensive assessment of air quality in a rapidly industrialized region experiencing significant urban growth. In contrast, the cities of Craiova and Drobeta-Turnu Severin also have different urban and industrial dynamics that, while important, require fewer sensors. For this paper, the sensors were chosen from two Romanian cities, Craiova and Drobeta-Turnu Severin, to cover the same surface at a 100 km distance. The first sensor from Adana, 20FDDC62, is located at 37.011 latitudes and 35.280 longitudes in the Yuregir region. The sensor 20FD2908 is outside of an apartment (37.0320 latitude and 35.302 longitude) in the Karaisalı area of Adana city. The third sensor has the ID 20FD51B8 (37.061 latitudes and 35.384 longitudes) and is in the Seyhan region. The sensor from Craiova has the ID 820002C3 (44.3194 latitudes and 23.8011 longitudes) and is on the university’s exterior wall in the city center. The last sensor from Drobeta-Turnu Severin has the ID 160002C2 (44.62383 latitudes and 22.640863 longitudes) and is on an exterior wall of a high school located near the Danube River and in a busy traffic area.

Adana is the sixth most significant and most rapidly developing area among 81 other regions of Turkey. It has agricultural, industrial, and trade potential. It has an urban population of 1.4 million. To evaluate the risk and effects of traffic-related pollution in Adana, some authors measured the metals accumulated in rosemary leaves along the highway [47]. Adana has a Mediterranean climate with long, hot summers and short, mild winters. During summer, temperatures usually peak in late July and August, with daytime temperatures exceeding 35 °C. Winter temperatures generally hover around 10–15 °C. Average monthly relative humidity in Adana typically ranges from 49% in August to 81% in January.

Craiova is the sixth most densely populated city in Romania and the most important city of the Oltenia region (SW part of Romania). Craiova has 243,765 inhabitants (census 2022). Craiova has a continental climate; cold, snowy, partly cloudy winters; and warm and generally clear summers. Drobeta-Turnu Severin has a temperate–continental climate with sub-mediterranean influences, with sunny and hot summers and mild winters. This city is in the western part of Oltenia and has 79,865 inhabitants (census 2022). In total, 30% of this number of inhabitants is represented by young (0–14 years) and older adults (>65), vulnerable categories to air pollution. Drobeta-Turnu Severin has a climate with sub-mediterranean influences, which is closer to that in Adana.

The sensors used in this study are the same type (PM Smoggie) with one exception, represented by the sensor with the ID 820002C3 (model A3) from Craiova. PM Smoggie measures two meteorological parameters: air temperature (0.5 °C resolution, ±1 °C accuracy) and relative humidity (1% resolution, ±2% accuracy) and PM_1_, PM_2.5_, and PM_10_ concentrations in the air (1 µg/m^3^ resolution, ±5% accuracy, and R^2^ = 0.99%, 81.6%, and 99.9% for all fractions’ coefficient of correlation to reference a gravimetric sampler) using an integrated laser-scattering detector [48,49,50]. PM Smoggie measures the meteorological parameters using microelectromechanical systems and the PM concentrations using a pulse of coherent IR light shining through a cavity with a PIN photodiode located sideways. A fan pushes the air into the chamber, and when a particle reaches the laser beam, it scatters the laser light. The PIN photodiode can detect scattered light. The number of events correlates to the mass concentration based on the proportionality relation between the amplitude of the recorded scattered signal and the particle size [51]. Sensor A3 is a bit more complex than PM Smoggie and can measure additional parameters like volatile organic compounds, carbon dioxide, formaldehyde, noise, and pressure. The additional parameters given by sensor A3 were not used in this study, only those common parameters measured by PM Smoggie (meteorological parameters and PMs concentrations). The measurement principles for meteorological parameters and PM concentrations are the same for PM Smoggie and A3.

The dataset includes three months (March, April, and May 2023) taken from three locations, one from Adana/Turkey and two from Romania. In total, 633,712 pieces of data were analyzed using the software SPSS Statistics version 23.0. These data are two meteorological parameters (temperature and relative humidity) and three particulate matter concentrations (PM_1_, PM_2.5_, and PM_10_). Each variable has several statistics associated with it.

We evaluated whether there was a significant difference between the two countries’ data, and the Mann–Whitney U test (also known as the Wilcoxon rank sum test) was applied [52]. This test determined whether the distributions of two independent (unrelated) groups were statistically significant.

## 3. Results and Discussion

Significant differences were observed in all five variables for Adana in Turkey and two cities in Romania: Drobeta-Turnu Severin and Craiova. For this reason, the measuring instruments in Adana/Turkey were first evaluated within themselves, and the measuring instruments in Romania were compared within themselves. Also, Table 1 displays the rankings for five parameters, temperature, relative humidity, and PM_1_, PM_2.5_, and PM_10_ concentrations, in three different locations within Adana/Turkey. The total number of data points collected was 391,396. Some sensors took measurements from Drobeta-Turnu Severin and Craiova (Romania). A total of 242,316 data were collected from these sensors. In Table 1, all five parameters of each location are presented separately. Considering the average of the 3-month data for Adana/Turkey, a temperature of 19.46 °C, humidity of 52.16%, PM_1_ of 6.82 µg/m^3^, PM_2.5_ of 11.14 µg/m^3^, and PM_10_ of 12.54 µg/m^3^ for are seen. For Drobeta-Turnu Severin and Craiova/Romania, these measurements made from two different locations show a value of 13.47 °C for temperature, 67.75% for humidity, 9.58 µg/m^3^ for PM_1_, 14.18 µg/m^3^ for PM_2.5_, and 15.79 µg/m^3^ for PM_10_.

**Figure 2:**
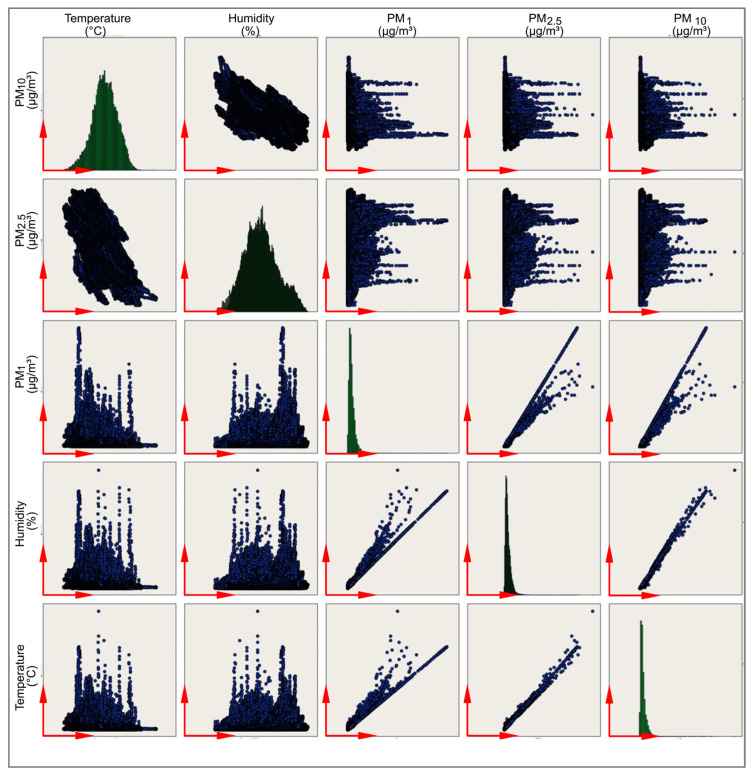
PM sensor scatterplot graphs of all the data.

The measurement results from sensors in Drobeta-Turnu Severin and Craiova/Romania show similar patterns to those observed in Adana/Turkey. Temperatures gradually increased from March to May, while humidity levels remained relatively stable. These measurements provide valuable data for analyzing environmental conditions and particulate matter concentrations in Adana/Turkey, Drobeta-Turnu Severin, and Craiova/Romania over a given period. Therefore, studying the correlation between these data allows us to determine whether there is a relationship between the variables, see Figure 2.

The correlation coefficients between temperature, humidity, PM_1_, PM_2.5_, and PM_10_ concentrations in Adana/Turkey are shown in Table 2. With a value of −0.617, the findings demonstrate a significant negative correlation between temperature and humidity. This finding suggests that humidity tends to decrease when temperature rises as well.

Furthermore, there are weak negative relationships between temperature and all three particle concentrations (PM_1_, PM_2.5_, and PM_10_). Lower particulate matter concentrations are related to higher temperatures. Also, there is a moderate positive correlation between humidity level and PMs. That is why, as relative humidity increases, the PM concentrations also tend to increase. Also, there are strong positive correlations between PM_1_, PM_2.5_, and PM_10_, emphasizing that different size ranges of PMs tend to be present at similar concentrations.

Table 3 provides the correlation coefficients to examine the relationships between temperature, humidity, and PM_1_, PM_2.5_, and PM_10_ concentrations in Drobeta-Turnu Severin and Craiova/Romania. Here, the results show a negative correlation between temperature and humidity with a coefficient of −0.515. This means that relative humidity tends to decrease as temperature increases. However, this correlation is relatively weaker than the results obtained for Adana/Turkey.

Also, weak and moderate negative correlations exist between temperature and PM_1_, PM_2.5_, and PM_10_. This result suggests that higher temperatures are associated with slightly lower concentrations of these categories of particulate matter. However, humidity does not show a significant relationship with particulate matter. In addition, there is a strong positive correlation between the correlation coefficients PM_1_, PM_2.5_, and PM_10_ in Drobeta-Turnu Severin and Craiova/Romania.

Overall, the correlation results for Drobeta-Turnu Severin and Craiova/Romania show a slightly different pattern than Adana/Turkey. While Adana/Turkey, Drobeta-Turnu Severin, and Craiova/Romania show negative correlations between temperature and particulate matter concentrations, the effect of relative humidity on particulate matter is noticeably different. In Adana, humidity has a moderate positive correlation with PMs, whereas, in Drobeta-Turnu Severin and Craiova/Romania, humidity shows little effect on PMs.

**Figure 3:**
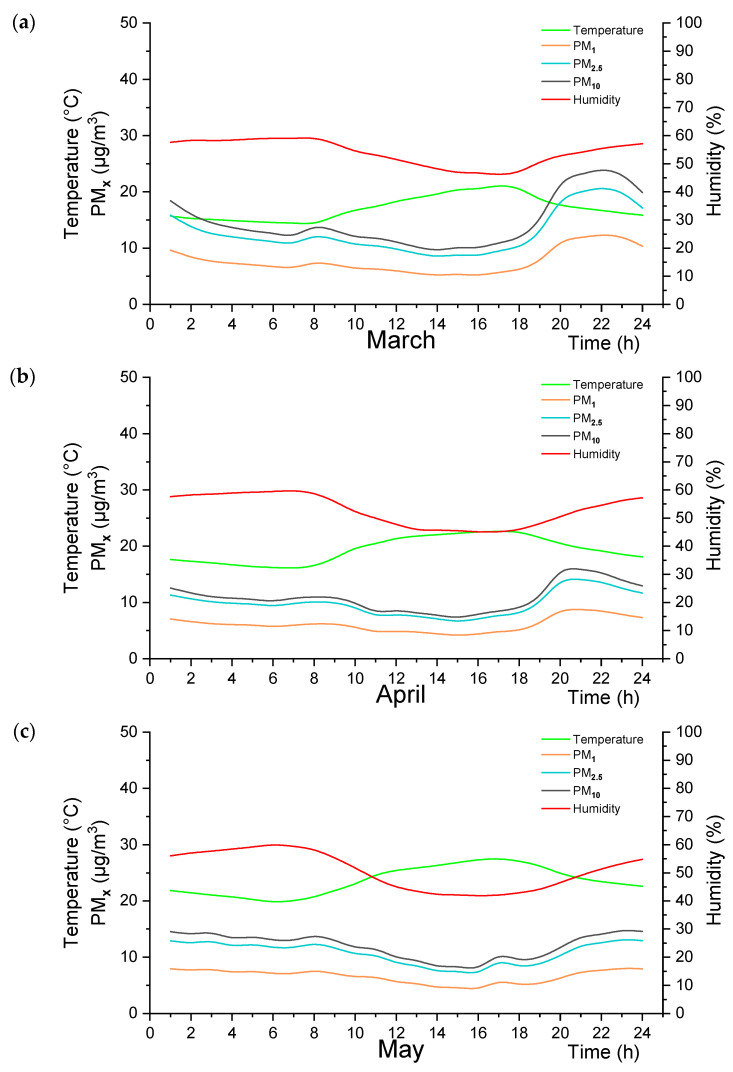
(**a**) Monthly average of the daily distributions for March, (**b**) April, and (**c**) May for Adana/Turkey.

**Figure 4:**
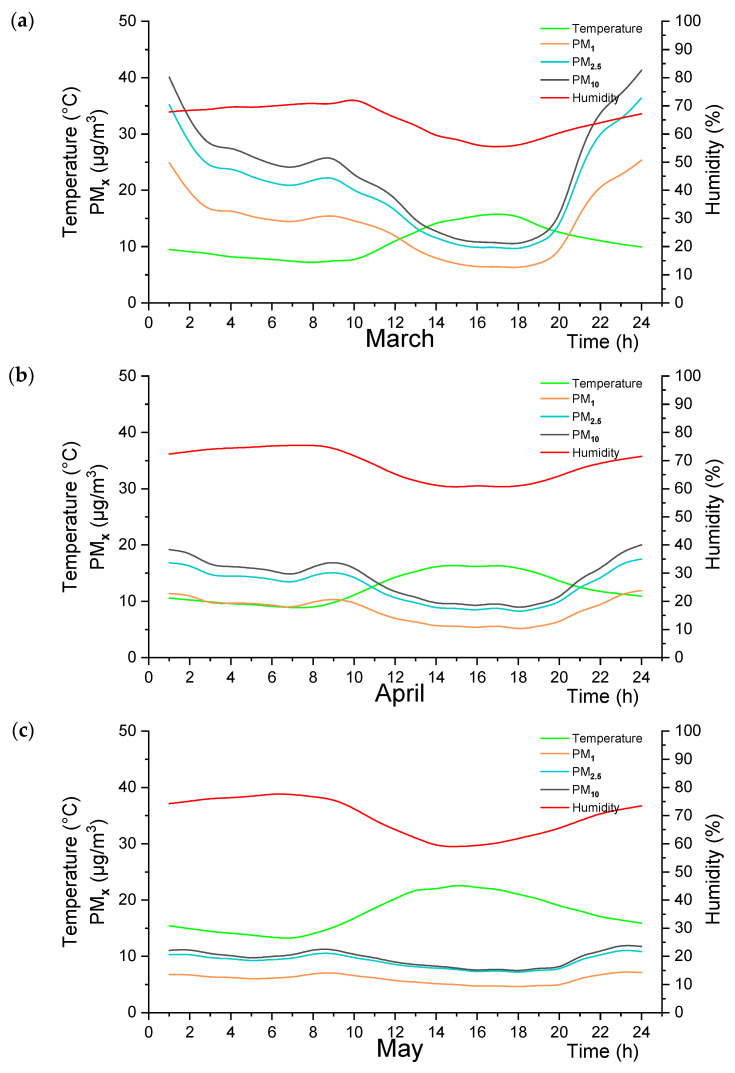
(**a**) Monthly average of the daily distributions for March, (**b**) April, and (**c**) May for Romania.

After the correlation results, the data obtained in one minute were analyzed hourly. Figure 3 took the average of three measurement sensors for Adana/Turkey. Figure 3a represents the results for March, Figure 3b illustrates the results for April, and Figure 3c represents the results for May. In Figure 4, for Drobeta-Turnu Severin and Craiova/Romania, hourly measurement results were shared by taking the average of the measuring instruments in two different cities. Figure 4a is the results of March, Figure 4b is the results of April, and Figure 4c is the results of May.

Figure 3a shows temperature ranges from 15.11 to 21.03 °C. The relative humidity levels range from 57.12% to 59.03%. Regarding air quality, the PM_1_ levels range from 7.03 to 12.30 µg/m^3^. The PM_2.5_ levels range from 11.13 to 20.58 µg/m^3^. The PM_10_ levels range from 12.38 to 23.83 µg/m^3^.

Figure 3b represents the statistical analysis of the measurement results for April in Adana/Turkey. Compared with March, there was a slight increase in temperature, ranging from 16.14 to 22.63 °C. The humidity levels remain relatively stable, ranging from 55.85% to 59.62%. Regarding air quality, there is a decrease in PM_1_ levels, ranging from 4.20 to 6.18 µg/m^3^. The PM_2.5_ levels also decrease, ranging from 6.74 to 10.09 µg/m^3^. The PM_10_ levels show a similar decrease, ranging from 7.41 to 10.98 µg/m^3^, compared to March results.

Figure 3c represents the statistical analysis of the measurement results for May in Adana/Turkey. Compared with April, there is a further increase in temperature, with a range of 19.55 to 27.46 °C. The humidity levels slightly decreased, ranging from 42.13% to 59.89%. Regarding air quality, there is an increase in PM_1_ levels, ranging from 4.58 to 7.70 µg/m^3^. The PM_2.5_ levels also increase, ranging from 7.43 to 13.05 µg/m^3^. The PM_10_ levels show a similar increase, ranging from 8.29 to 14.69 µg/m^3^.

Overall, the temperature gradually increases from March to May, while the humidity levels remain relatively stable. Regarding air quality, there is a slight decrease in PM_1_, PM_2.5_, and PM_10_ levels from March to April, followed by an increase from April to May. These findings suggest that there may be seasonal variations in air pollution in Adana/Turkey.

Figure 4a presents the statistical analysis of the measurement results for March in Drobeta-Turnu Severin and Craiova/Romania. It shows the time, temperature, humidity, and concentration levels of PM_1_, PM_2.5_, and PM_10_ particles. The temperature ranges from 7.98 to 15.75 °C. The humidity ranges from 55.52% to 70.84%. The concentration levels of PM_1_ particles range from 6.34 to 25.36 µg/m^3^. The concentration levels of PM_2.5_ particles range from 9.71 to 36.41 µg/m^3^. The concentration levels of PM_10_ particles range from 10.61 to 41.28 µg/m^3^.

Figure 4b shows the statistical analysis of the measurement results for April in Drobeta-Turnu Severin and Craiova/Romania. It offers similar information to the first table but for April. The temperature ranges from 8.91 to 16.33 °C. The humidity ranges from 60.68% to 75.34%. The concentration levels of PM_1_ particles range from 5.17 to 11.92 µg/m^3^. The concentration levels of PM_2.5_ particles range from 8.26 to 17.48 µg/m^3^. The concentration levels of PM_10_ particles range from 8.96 to 20.01 µg/m^3^.

Figure 4c represents the statistical analysis of the measurement results for May in Drobeta-Turnu Severin and Craiova/Romania. It shows similar information to the previous tables but for May. The temperature ranges from 14.02 to 22.56 °C. The humidity ranges from 59.02% to 77.56%. The concentration levels of PM_1_ particles range from 4.64 to 7.17 µg/m^3^. The concentration levels of PM_2.5_ particles range from 7.19 to 10.99 µg/m^3^. The concentration levels of PM_10_ particles range from 7.48 to 11.85 µg/m^3^.

The PM_1_, PM_2.5_, and PM_10_ values in Adana/Turkey vary for three months. In March, the PM_1_ values range from 7.03 to 24.91, steadily declining. April and May show lower PM_1_ values, showing relatively better air quality during these months.

When looking at the data from Romania, a different pattern is observed. There is a gradual increase in temperature from March to May, as in Adana/Turkey. However, relative humidity levels in Drobeta-Turnu Severin and Craiova/Romania show a more marked decrease over the three months. Looking at the PM values in Drobeta-Turnu Severin and Craiova/Romania, a similar trend is seen for Adana/Turkey, and the values generally decrease over time. For example, March has the highest PM_1_ values, ranging from 6.33 to 25.36, while May has the lowest values from 4.64 to 7.13. This indicates an improvement in air quality as the months progressed.

Some patterns can be read when we compare Adana/Turkey and Drobeta-Turnu Severin and Craiova/Romania measurements. Both locations show a decrease in PM values over time. This result is evidence of a gradual improvement in air quality. However, we can state that the reduction in Drobeta-Turnu Severin and Craiova/Romania is higher than in Adana/Turkey and can be attributed to various factors, such as different sources of pollution and weather conditions in the two regions. It can be observed that some curved trends regarding hourly changes in PM values. In both places, PM values tend to be higher early in the day (e.g., 1–4 in the morning) and lower from late morning to early evening. This model shows that pollutant emissions may be higher at night and early in the morning and gradually decrease as the day progresses.

As it is known, the presence of PM in the air causes adverse effects on human health. However, when we look at the studies in the literature and as seen in this study, PM concentration is affected by various meteorological conditions such as air temperature, relative humidity, wind speed, and precipitation. In this context, in addition to the primary analysis of this study, a comparative study on PM concentrations in the atmosphere of Adana, Drobeta-Turnu Severin, and Craiova was conducted, and the findings were juxtaposed with similar studies. Yousefian et al. (2020) [53] used a longer dataset provided by the National Environment Agency, covering a wider range of pollutants. Tehran, the capital city, experiences heavier traffic and different pollution dynamics compared to Craiova, Drobeta-Turnu Severin, and Adana. This study found a significant correlation between temperature and these particles, with different seasonal and “weekend effects” on PM_2.5_ and PM_10_ levels, a phenomenon only partially reflected in the current study. The study by Shikhovtsev et al. (2023) [54] in the Southern Baikal Region highlights the influence of topography, meteorological conditions, and regional atmospheric circulation on air pollution. Similar to the current study, higher PM_2.5_ and PM_10_ concentrations and different seasonal patterns were recorded in urban areas in this study. In particular, the impact of forest fires in summer and the burning of solid fossil fuels in thermal power plants in winter were identified as the main sources of pollution.

According to the statistical analysis of the effect of PM_10_ concentrations on air temperature [55], a strong correlation was observed between the temperature and PM_10_ due to the measurements made in the Caribbean Basin. A good correlation (−0.553) between PM_10_ and temperature was found in Craiova by Udristioiu et al. (2023) [56,57] using an independent sensor network as an alternative to the governmental network for the citizens’ benefit. Moreover, the independent sensor network agreed with those from the official monitoring network and produced data when the official monitoring had missing values. It has been analyzed that this correlation is stronger, especially between May and September. In the relationship between surface temperature and PM concentration in a long-term observational data study conducted by Kim, M (2019) [58] in Seoul, the observations made over ten years underlined a strong positive correlation. According to the study by Zoran et al. (2020) [13] in Milan, Italy, during COVID-19, PM concentrations were positively correlated with mean surface air temperature and inversely correlated with air relative humidity. Therefore, the increase in daily COVID-19 new cases was found to be positively correlated with PM and Air Quality Index.

There is a correlation between PM concentration and meteorological factors, but the strength and direction of the correlation varies with location, climate, and time zone. According to the temperature and humidity effects on particulate matter concentrations, research was conducted in New Zealand [59], which has a sub-tropical climate; there is a negative correlation between humidity and PM_10_. In addition, the relationship between PM and meteorological factors also varies over time, possibly due to climate variability and changes in global weather patterns, according to the review study conducted by Tanatachalert and Jumlongkul (2023) [60]. In 2023, it was emphasized that the relationship between relative humidity and particulate matter is essential.

Seasonal variations are observed in PM concentration, with higher concentrations typically seen during winter. According to the results of PM samples obtained by Nguyen et al. (2017) [61]. In Korea, with a one-year sampling, it was observed that the average concentrations of PM_1_, PM_2.5_, PM_2.5–10_, and PM_10_ were lowest in summer and highest in winter. In addition, according to the study conducted by Zhao et al. (2014) [62] in Beijing, it was concluded that wind speed and relative humidity are the two main factors affecting the distributions of PM_2.5_ and PM_10_ concentration. As stated in the studies mentioned above, there is a strong correlation between air pollution and meteorological factors. However, more research is needed, including advanced analytical techniques and modeling, to better understand the mechanisms and effects of air pollution.

## 4. Conclusions

The statistical analysis of air quality measurements in Adana/Turkey, Drobeta-Turnu Severin, and Craiova/Romania reveals some interesting trends. In both countries, air quality improved over time in March, April, and May, and PM values were analyzed to decrease in general. Especially in March, due to the necessity of meeting the heating needs and the low-temperature values, it is predictable that PM will be higher. Hourly variations indicate higher PM values in the early morning and lower values during the daytime. The concentrations of particulate matter are negatively correlated with temperature in Adana/Turkey, Drobeta-Turnu Severin, and Craiova/Romania; the effect of humidity on particulate matter is significantly different. In Adana, humidity has a moderately positive correlation with particulate matter, while in Romania, relative humidity has little consistent effect on PM_2.5_ and PM_10_. However, there appears to be a strong correlation between PMs.

This study provides solid evidence that immediate action is required to address air pollution and its detrimental impacts on human health by carefully analyzing quarterly data about meteorological conditions and particulate matter concentrations. These invaluable results deserve to be widely disseminated and serve as a crucial reference for future research efforts in air pollution and its multifaceted impacts.

Remarkably, the innovative modeling approach outlined in this study shows promise beyond the regions analyzed. The novel model concept presented here can be applied in areas where observation stations are not available. Thus, the modeling practice here will significantly benefit local communities in modeling environmental pollution. Considering the future decline in low air quality, the model work here can provide useful predictions, especially for public agencies and local communities. The final findings presented in this paper highlight the crucial need for an urgent and comprehensive response to air pollution, and these findings deserve to serve as an essential reference for all future research initiatives aimed at tackling the profound effects of air pollution.

## Figures and Tables

**Table 1:** The mean and median measurement results for Adana/Turkey and Drobeta-Turnu Severin and Craiova/Romania.

Countries	Sensors	Months	Mean/Median	Temp. (°C)	Relative Humidity (%)	PM_1_ (µg/m^3^)	PM_2.5_ (µg/m^3^)	PM_10_ (µg/m^3^)
Adana/ Turkey	1	March	Mean	19.46	52.16	4.93	8.10	8.82
			Median	19.18	53.50	4.00	6.00	7.00
		April	Mean	22.52	49.27	4.22	6.78	7.24
			Median	22.55	49.00	3.00	6.00	6.00
		May	Mean	25.40	49.04	5.59	9.16	10.00
			Median	25.56	49.50	5.00	8.00	9.00
	2	March	Mean	16.35	51.07	9.09	14.92	17.90
			Median	15.94	53.00	8.00	13.00	15.00
		April	Mean	18.36	50.71	7.32	11.66	13.32
			Median	17.40	53.50	7.00	11.00	12.00
		May	Mean	22.84	48.69	7.68	12.32	14.39
			Median	22.40	50.00	8.00	12.00	14.00
	3	March	Mean	15.55	58.75	9.10	15.24	17.21
			Median	15.76	60.00	8.00	13.00	14.00
		April	Mean	17.48	58.29	6.60	10.78	11.64
			Median	17.07	59.50	6.00	10.00	10.00
		May	Mean	22.52	54.76	6.50	10.76	11.68
			Median	22.10	55.50	6.00	10.00	11.00
Drobeta-Turnu Severin and Craiova/ Romania	1	March	Mean	14.01	63.18	13.18	22.14	25.67
			Median	13.20	52.70	11.00	19.00	21.00
		April	Mean	14.41	92.20	8.36	14.32	16.16
			Median	13.90	59.70	7.00	13.00	13.00
		May	Mean	19.58	60.00	6.89	11.47	12.15
			Median	18.70	61.70	6.00	11.00	11.00
	2	March	Mean	7.86	76.49	15.10	19.15	21.30
			Median	7.82	76.90	12.00	15.00	17.00
		April	Mean	10.16	78.71	8.55	11.10	12.14
			Median	9.74	79.40	6.00	8.00	9.00
		May	Mean	15.35	79.17	5.13	6.92	7.35
			Median	14.92	79.40	4.00	6.00	6.00

**Table 2:** Correlation results for Adana/Turkey.

Adana/Turkey	Temperature (°C)	Humidity (%)	PM_1_ (µg/m^3^)	PM_2.5_ (µg/m^3^)	PM_10_ (µg/m^3^)
Temperature	1	−0.617 **	−0.167 **	−0.173 **	−0.165 **
Humidity	−0.617 **	1	0.337 **	0.360 **	0.347 **
PM_1_	−0.167 **	0.337 **	1	0.981 **	0.968 **
PM_2.5_	−0.173 **	0.360 **	0.981 **	1	0.990 **
PM_10_	−0.165 **	0.347 **	0.968 **	0.990 **	1

** Correlation is significant at the 0.01 level (2-tailed).

**Table 3:** Correlation results for Drobeta-Turnu Severin and Craiova/Romania.

Drobeta-Turnu Severin and Craiova/Romania	Temperature (°C)	Humidity (%)	PM_1_ (µg/m^3^)	PM_2.5_ (µg/m^3^)	PM_10_ (µg/m^3^)
Temperature	1	−0.515 **	−0.241 **	−0.184 **	−0.193 **
Humidity	−0.515 **	1	0.113 **	−0.015 **	−0.008 **
PM_1_	−0.241 **	0.113 **	1	0.979 **	0.979 **
PM_2.5_	−0.184 **	−0.015 **	0.979 **	1	0.998 **
PM_10_	−0.193 **	−0.008 **	0.979 **	0.998 **	1

** Correlation is significant at the 0.01 level (2-tailed).

## Data Availability

The data presented in this study are shown in the paper.
